# Association between the CCR5Δ32 polymorphism and preeclampsia

**DOI:** 10.1186/1753-6561-8-S4-P70

**Published:** 2014-10-01

**Authors:** Bianca de Paula Telini, Tiago Degani Veit, Priscila Vianna, José Artur Bogo Chies

**Affiliations:** 1 Universidade Federal do Rio Grande do Sul, Porto Alegre, Brazil

## Background

Preeclampsia (PE) is a condition that occurs in up to 7% of all pregnancies. Its exact pathophysiology is not known yet, but there is the involvement of genetic and immune factors maternal and fetal, with occurrence of hypertension and proteinuria. There is evidence of increased systemic inflammation during the first trimester of pregnancy in women with preeclampsia. The PE usually develops in the second half of pregnancy and is characterized by events of endothelial dysfunction and inflammation in its pathogenesis. Chemokines (proinflammatory cytokines) are considered the main determinants of the inflammatory response and its action by binding to specific receptors can be directly related to the development of PE. The chemokine receptor type 5 (CCR5) is a protein encoded by the CCR5 gene which is located on chromosome 3p21.3 p24. The polymorphic variant CCR5delta32 resulting from deletion of 32 base pairs in this gene leads to production of a non-functional isoform of the receptor, and has been implicated in a variety of autoimmune diseases.

## Methods

To investigate the role of this polymorphism in the pathogenesis of preeclampsia, we evaluated the frequency of polymorphic variant CCR5Δ32 among women with and without PE. In order to do this, 155 pregnant women with PE and 144 pregnant women without PE were genotyped (both groups with similar age).

## Results and conclusions

The presence of the mutant delta 32 was associated to protection against the development of PE, i.e. carriers of the Δ32 allele had a lower chance of developing preeclampsia (OR 0.342, CI 95%, p = 0.047). We suggest that cells involved in the modulation of the immune response during pregnancy which express this protein cannot migrate properly to certain sites of inflammation and this could alter the individual's inflammatory profile. Such an imbalance would make less likely the establishment of a pathological inflammatory profile characteristic of preeclampsia. Our study suggests the CCR5Δ32 as an independent factor in the susceptibility to PE together with primary hypertension, which was also associated to PE risk in our cohort (OR 8.696, CI =95%, p < 0.001).
